# The Association Between Urinary Concentrations of Organophosphate Metabolites and Asthma-Related Outcomes Among Schoolchildren From Informal Settlements

**DOI:** 10.3389/ijph.2023.1606174

**Published:** 2023-08-22

**Authors:** Mashudu Mthethwa, Mohamed Jeebhay, Toyib Olaniyan, Lubbe Wiesner, Bhawoodien Parker, Joy Leaner, Martin Röösli, Mohamed Aqiel Dalvie

**Affiliations:** ^1^ Centre for Environmental and Occupational Health Research, School of Public Health, Faculty of Health Sciences, University of Cape Town, Cape Town, South Africa; ^2^ Division of Occupational Medicine, University of Cape Town, Cape Town, South Africa; ^3^ Division of Clinical Pharmacology, Department of Medicine, University of Cape Town, Cape Town, South Africa; ^4^ Department of Environmental Affairs and Developmental Planning, Western Cape Government, Cape Town, South Africa; ^5^ Swiss Tropical Public Health Institute, Allschwil, Switzerland; ^6^ University of Basel, Basel, Switzerland

**Keywords:** pesticides, organophosphates, informal settlements, child asthma, urinary dialkyl phosphates

## Abstract

**Objectives:** There is inconsistent evidence on the relationship between pesticide exposure and childhood respiratory outcomes in non-agricultural settings. This study investigated the association between organophosphate (OP) pesticide exposure and asthma-related outcomes in children residing in four informal settlements.

**Methods:** The study was a longitudinal study of 590 schoolchildren, with a 12 months follow-up period. A standardised questionnaire adopted from the International Study of Asthma and Allergies in Childhood was administered to caregivers for child’s respiratory symptoms and household characteristics. Spirometry and fractional-exhaled nitric oxide, including a phadiatop test (atopy status) and urinary dialkyl phosphate (DAP) metabolites were measured at baseline and follow-up. DAP metabolites included diethylphosphate (DEP) and dimethyl phosphate (DMP) measured at baseline and follow-up and dimethylthiophosphate (DMTP) measured only at baseline.

**Results:** The mean ages of schoolchildren were 9.9 ± 0.91 years and the overal incidence proportions of new doctor diagnosed asthma was 2.2%. No consistent patterns of increased risk of asthma outcomes with increasing DAP concentrations was found in multivariate analysis.

**Conclusion:** Future studies with longer follow-up periods and repeated OP biomonitoring are recommended.

## Introduction

According to the Global asthma report 2018, South Africa ranks 5th in asthma mortality worldwide [1]. The asthma prevalence among 13–14 years-old in Cape Town, South Africa (SA) was 20% in 2002 [2]. Furthermore, 47% of asthmatic children living in Cape Town were undiagnosed especially in areas of low socioeconomic status [3].

Studies in both children and adults have found evidence of an association between different types of pesticides, including commonly used organophosphates, with asthma-associated outcomes [4–9]. However, these studies have primarily been conducted in rural areas.

In non-agricultural settings, pesticide exposure can occur from household use. In urban South Africa, particularly in informal settlements, illegal highly concentrated pesticides intended for agriculture labelled as domestic pesticides, are widely and easily accessible from informal markets [10]. These pesticides include organophosphates (e.g., chlorpyrifos and methamidiphos), pyrethroids (e.g., cypermethrin) and carbamates (e.g., aldicarb). Poor living conditions often provide breeding grounds for pests in informal settlements and may lead to high residential utilization of these street pesticides [10]. Children are especially more susceptible to these exposures due to developmental and physiological factors [11]. Children residing in urban and informal settlements are considerably exposed to residential pesticides [11].

Very little information exists on residential or household pesticides exposures and respiratory outcomes in children especially in non-rural settings. Three studies conducted in non-rural settings of the United States (US) [4, 12, 13] and one in a farming and a non-farming setting in Lebanon [14] found an association between reported residential pesticide use and increased respiratory symptoms. Pearla et. al. [15] found no association between respiratory symptoms and DAP metabolites in a US population based study. The current study sought to investigate the associations between OP pesticides exposure and asthma-related outcomes among children residing in selected informal settlements in the urban and rural Western Cape, SA.

## Methods

### Study Design and Population

This 12 months longitudinal analysis on the association between OP pesticide exposure and asthma-related outcomes, made use of data from a larger study investigating the association between air pollution and asthma-related outcomes with baseline data collection conducted between February 2015 and August 2015 [16]. A total of 600 children were recruited from six primary schools located in informal settlements from four areas including an urban industrialised area (Marconi Beam), a peri-urban area (Khayelitsha), a rural area (Oudtshoorn) and an urban low-industrialised area (Masiphumelele) in the Western Cape. Follow-up data was collected between May 2016 and September 2016, with each schoolchild (hereafter, “learner”) sampled at approximately 12 months after baseline data collection. Grade 4 learners were selected to participate in the study because at their age, they were able to cooperate with the instructions of the study and would not graduate to secondary school before the study was completed, thereby minimizing any loss of follow-up data. A total of 150 learners were targeted from each of the four study areas for a study sample of 600 (as determined by sample size calculations). After approval from the school principal and the school board at each school, a list of Grade 4 learners was obtained, including their addresses to acquire parental/guardian consent. Random sampling was used to select 150 parental-consented participants from the entire Grade 4 list per study area at baseline. However, due to lower participation in the urban low-industrialised area, additional participants were recruited opportunistically from the peri-urban and rural areas. Details of the sampling is described elsewhere [16]. The exclusion criteria were as follows:

i) Non-grade 4 learner;
ii) learner had a recent operation (in last 12 months);
iii) learner has any pain or nausea;
iv) learner is being treated for Tuberculosis;
v) learner had flu, sinusitis or lung infection in the last 3 weeks;
vi) learner has a history of epilepsy.

The overall participation rate for the study was 95%. A small proportion of participants (<11%) that participated at baseline did not participate at follow-up and a small proportion of participants did not provide urine samples at baseline and follow-up (<10%).

The communities were engaged in the design and implementation of the study through meetings held at the schools with parents and learners and through stakeholder meetings that included the members of the communities, community leaders and non-governmental organisations in each study area during the design and implementation of the study.

### Questionnaire Data

A standardised questionnaire, adopted from the International Study of Asthma and Allergies in Childhood (ISAAC), was administered to caregivers.

Information about the child on previous rhinitis, doctor-diagnosed asthma, ocular-nasal symptoms, wheezing and other respiratory symptoms, demographic information, host characteristics, and indoor exposures (including household pesticide use) were obtained. Questionnaires were administered by trained staff in the caregiver’s preferred language. The age, gender, height and weight of each learner were obtained when the lung function test was conducted at the schools selected in the four study areas.

An asthma symptom score was computed from a sum of 8 asthma-associated outcomes (doctor diagnosed asthma, asthma medication use, wheeze and breathlessness, chest-tightness, shortness of breath while walking, woken up by attack of shortness of breath, coughing with exercise and wheezing), using a cut-off score of two or more symptoms [17]. New-onset of asthma-related reported symptoms were defined as the proportion of new symptoms reported by the learner at 12 months follow-up amongst those learners who were symptom-free during the baseline study.

### Pesticide Biomonitoring

Spot urine samples were collected at school during both the baseline and follow-up data collection phases by trained research assistants in colourless 50 mL plastic urine containers, sealed with plastic caps and stored at −20°C before being analysed by the Division of Clinical Pharmacology Laboratory at the University of Cape Town (UCT). Three Dialkyl phosphate (DAP) metabolites including diethylphosphate (DEP), dimethylthiophosphate (DMTP) and dimethyl phosphate (DMP) were measured as no standards were in stock at the laboratory for the other metabolites. However, the measured DAPs are the most frequently detected and the commonly measured in literature and covered the full spectrum of DAP producing OP pesticides [18]. DEP and DMP were measured at baseline and follow-up and DMTP only at baseline (no standards were in stock at the analytical laboratory at follow-up). ΣDAP therefore was therefore calculated differently at baseline compared to follow-up).

Analysis and quantification of three urine DAP metabolites was done using a validated high-performance liquid chromatography with tandem mass spectrometer detection (LC-MS/MS) assay developed at the Division of Clinical Pharmacology Laboratory at UCT. The limit of quantification (LOQ) value for all three DAP metabolites during measurement was 1.56 ng/mL. Values that were below the LOQ were transformed by dividing 1.56 by the square root of 2 (1.56/√2).

### Allergy Tests

Blood samples were drawn using a Becton Dickson Vacutainer serum separating tube (Becton, Dickson and Company Vacutainer Systems, Oxford, United Kingdom) and kept in a 4°C cooler box before being transported to the laboratory for centrifugation. The blood tubes kept at room temperature for 1–2 h to allow for the blood to clot, followed by centrifugation at 1,350 rpm for 10 min. The serum was stored at −20°C before being transported to the National Institute for Occupational Health (NIOH) immunology laboratory in Johannesburg for testing. A Phadiatop test (Phadia AB, Uppsala, Sweden) was used to determine the presence of sensitization to common aeroallergens such as house dust mites, grass pollens, cat, dog, and cockroach. The test was conducted at both baseline and follow-up.

### Spirometry

Forced vital capacity (FVC) and forced expiratory volume in one second (FEV_1_), were assessed using spirometry by a trained nurse during school visits at baseline and at follow-up studies, according to the American Thoracic Guidelines [19]. The procedure is described elsewhere [16]. Airway obstruction was characterised as FEV_1_ less than 80% of the predicted value, values less than the lower limit of normal (LLN), and an FEV_1_/FVC ratio less of than 0.8. Learners who did not had airway obstruction at the baseline study were classified as new cases, when it became evident that they had developed airway obstruction at the 12 months follow-up study.

### Forced Exhaled Nitric Oxide (FENO)

FeNO was conducted by a trained nurse to assess allergic airway inflammation at both baseline and follow up using a handheld portable nitric oxide sampling device (NIOX MINO^®^ Airway Inflammation Monitor (NIOX MINO; Aerocrine AB, Solna, Sweden), while learners were in a sitting position [15]. Measurements were taken according to the American Thoracic Society/European Respiratory Society recommendations [20]. Measurements for learners with upper and lower respiratory tract infection were postponed and recorded once they have recovered. Ambient Nitrogen Oxide (NO) was also recorded. Airway inflammation was defined as a FeNO > 35 ppb. Learners who did not have airway inflammation at the baseline study were classified as new cases if they developed airway inflammation at the 12 months follow-up study.

### Statistical Analyses

Data analyses were conducted using the STATA statistical software package version 15. All continuous data that were not normally distributed were summarized using median and interquartile ranges, and log transformed using the natural log of values of the variables for further analysis. Univariate and multivariable linear regression were conducted to assess the associations between exposure variables and continuous asthma-associated outcomes such as FEV_1_, FVC and FEV_1_/FVC ratio. Univariable and multivariable logistic regression models were done to assess the associations between exposure variables and binary outcomes, including doctor diagnosed asthma, asthma symptom score, rhinitis, airway obstruction and airway inflammation. Cross-sectional associations were investigated at baseline and 12 months follow-up and longitudinal associations were investigated between average concentrations of DAPs (for baseline and follow-up) and newly defined outcomes at 12 months, amongst participants without the outcome at baseline. A stratified analysis was conducted to assess effect-modification by gender and atopic status. The models were adjusted *a priori* for known confounders including individual host characteristics: age, gender, body mass index, maternal smoking, birthweight, atopy, smokers in the house, and environmental pollutants such as annual ambient air pollutants [Nitrogen Dioxide (NO_2_) and fine particular matter ≤ 2 μm (PM_2.5_)], and study area that were found to be significant in the main study [20]. Study areas were added to the models to account for any variations in area-specific characteristics. Regression coefficients and odds ratios were interpreted as change per ng/mL increase in DAP metabolite concentrations. Additionally, a linear mixed-effect model including both the baseline and follow-up continuous outcomes with random effects for participant, and fixed effects for the exposure and confounder variables were conducted, accounting for repeated measures. Quintiles were used in categorising DAP concentrations. The lowest quintile was used as the reference category. Restricted cubic splines were created to account for the non-linear relationship of the continuous covariates [such as age, body mass index (BMI), child birthweight, annual PM_2.5_, and annual NO_2_] with the outcome of interest.

### Ethics

Ethics approval of the main study (HREC Ref: 697/2014), as well as the sub-study (HREC Ref: 597/2020) was granted by the Health Sciences Research Committee of the University of Cape Town. Approval to conduct research at primary schools was obtained from the Western Cape Department of Education (Reference number: 20140917-36653) during the main study. Informed consent was obtained from parents and legal guardians, and children assented to participating in the study before commencement of all tests.

## Results

### Descriptive Demographic, Host Characteristics and Indoor Household Exposures

The mean age was similar and ranged from 9.6 to 11.5 years across all four study areas (Table 1). There was also an approximate equal distribution in gender across the four study areas. The mean BMI was significantly higher at follow-up compared to baseline (18.8 vs. 17.7 kg/m^2^, *p < 0.05*). Children residing in the rural area had the lowest prevalence of atopy (28.1%) as compared to the other three study areas (average prevalence = 40%). The prevalence of maternal smoking was lower in the urban low-industrialised and urban industrialised areas than in the other two study areas. Furthermore, the prevalence of self-reported pesticide-use was significantly lower at baseline (9%) compared to follow-up (15.5%). Conversely, the prevalence of reported pet ownership and presence of smokers in the household were higher at baseline compared to the follow-up study (Table 1).

**TABLE 1:** Demographic, host factors, and indoor household exposures of learners living in informal settlements of the Western Cape Province during the baseline (2015) and follow-up (2016) studies (The Association Between Urinary Concentrations of Organophosphate Metabolites and Asthma-Related Outcomes Among Schoolchildren From Informal Settlements, Western Cape, South Africa. 2015–2016).

Variable	Marconi-beam urban industrialised		Masiphumelele urban low-industrialised		Khayelitsha peri-urban		Oudtshoorn rural		All areas	
	Baseline	Follow-up	Baseline	Follow-up	Baseline	Follow-up	Baseline	Follow-up	Baseline	Follow-up
	*N* = 150	*N* = 136	*N* = 117	*N* = 109	*N* = 163	*N* = 131	*N* = 170	*N* = 159	*N* = 600	*N* = 535
Age (years)	10.3 ± 0.92	11.3 ± 0.93	10.4 ± 0.94	11.5 ± 0.95	9.6 ± 0.74	10.6 ± 0.75	9.6 ± 0.76	10.6 ± 0.76	9.9 ± 0.91	10.9 ± 0.92
Gender, female	74 (49.3)		49 (41.8)		83 (54.3)		92 (54.1)		298 (50.5)	
Weight (kg)	34.3 ± 7.7	40.1 ± 10.2	35.0 ± 9.2	40.0 ± 11.1	31.9 ± 7.1	37.0 ± 8.8	28.5 ± 5.6	33.9 ± 8.0	**32.2 ± 7.8**	**37.6 ± 9.8**
Height (cm)	137.6 ± 6.8	142.5 ± 6.9	137.1 ± 7.2	142.2 ± 8.5	131.8 ± 8.8	139.1 ± 7.3	132.2 ± 6.0	140.2 ± 6.7	**134.4 ± 7.7**	**140.9 ± 7.5**
BMI (kg/m^3^)	17.9 ± 3.1	19.5 ± 3.7	18.4 ± 3.9	19.5 ± 4.0	18.5 ± 4.2	19.0 ± 3.6	16.2 ± 2.4	17.1 ± 2.9	**17.7 ± 3.5**	**18.8 ± 3.8**
Atopy	61 (43.6)	—	47 (42.3)	—	61 (41.5)	—	46 (28.2)	—	215 (38.3)	—
Birthweight <2.5 kg	4 (2.7)	—	2 (1.8)	—	16 (9.9)	—	24 (14.3)	—	46 (7.8)	—
Maternal smoking	21 (14.0)	—	13 (11.8)	—	35 (21.6)	—	39 (23.2)	—	108 (18.3)	—
Pesticide use	8 (5.3)	31 (22.8)	14 (12.9)	40 (36.7)	11 (6.8)	22 (16.8)	20 (11.9)	0	**53 (9.0)**	**93 (15.5)**
Visible mould	5 (3.3)	15 (11.0)	17 (15.6)	8 (7.4)	11 (6.8)	7 (5.3)	19 (11.4)	1 (0.3)	52 (8.8)	31 (5.6)
Dampness	7 (4.7)	12 (8.8)	15 (13.9)	14 (12.8)	8 (4.9)	9 (6.9)	12 (7.2)	0	42 (7.2)	35 (6.5)
Pet ownership	15 (10.0)	9 (6.6)	12 (11.1)	6 (5.5)	47 (29.0)	10 (7.6)	43 (25.8)	16 (10.1)	**117 (19.9)**	**41 (7.7)**
Smoker at home	26 (17.3)	21 (15.4)	34 (31.2)	25 (22.9)	49 (30.3)	27 (20.6)	55 (32.9)	23 (14.5)	**164 (27.9)**	**96 (17.9)**
Paraffin use	112 (74.1)	79 (72.5)	67 (62.0)	97 (71.3)	63 (38.9)	97 (71.3)	85 (50.9)	4 (2.5)	327 (55.7)	276 (51.6)
Annual PM_2.5_	10.7 (8.9–13.2)						6.9 (5.6–8.7)		12.3 (10.3–13.8)	
Annual NO_2_	23.1 (21.7–24.4)						7.9 (6.9–8.9)		23.8 (22.7–24.9)	

Continuous data presented as mean ± SD/median (IQR), categorical data as N (%). Bold figures indicate statistical significance between baseline and follow-up (*p* < 0.05).

### Parental-Reported Asthma Associated Outcomes

At baseline, children from the urban low-industrialised area had a high 12 months prevalence of reported rhinitis (36.2%) and wheezing (23.6%) compared to the other study areas, while the prevalence of asthma symptom score ≥ 2 (based on the sum of positive answers (yes = 1) to 8 main asthma symptoms and bronchial hyper-responsiveness questions) was highest in children residing in the urban industrialised (10.4%) and urban low-industrialised (10%) areas (Table 2). This scenario changed at follow-up with the 12 months prevalence of rhinitis (20.1% vs. 3.6%, *p < 0.05*) and wheezing (12.9% vs. 4.9%, *p < 0.05*) dropping significantly across the four areas while the 12 months prevalence of ocular-nasal symptoms (25.6% vs. 32.1%, *p < 0.05*) and asthma-symptom scores ≥ 2 (7% vs. 17.9%, *p < 0.05*) increased across the four areas.

**TABLE 2:** Parental-reported asthma-associated outcomes among learners living in the informal settlements of Western Cape at the baseline (2015) and follow-up (2016) studies (The Association Between Urinary Concentrations of Organophosphate Metabolites and Asthma-Related Outcomes Among Schoolchildren From Informal Settlements, Western Cape, South Africa. 2015–2016).

Variable	Marconi-beam urban industrialised		Masiphumelele urban low-industrialised		Khayelitsha peri-urban		Oudtshoorn rural		All areas	
	Baseline	Follow-up	Baseline	Follow-up	Baseline	Follow-up	Baseline	Follow-up	Baseline	Follow-up
	*N* = 150	*N* = 136	*N* = 117	*N* = 109	*N* = 163	*N* = 131	*N* = 170	*N* = 159	*N* = 600	*N* = 535
Ocular-nasal symptoms	29 (19.3)	44 (32.4)	29 (26.4)	61 (55.9)	48 (29.6)	44 (33.6)	25 (26.8)	20 (12.6)	**151 (25.6)**	**175 (32.1)**
Rhinitis	41 (27.3)	2 (1.5)	39 (36.1)	8 (7.3)	12 (7.4)	9 (6.9)	26 (15.6)	0	**118 (20.1)**	**19 (3.6)**
Wheezing	17 (11.3)	9 (6.6)	26 (23.6)	5 (4.6)	12 (7.4)	9 (6.9)	21 (12.5)	1 (0.6)	**76 (12.9)**	**24 (4.5)**
Doctor diagnosed asthma	4 (2.7)	2 (1.5)	4 (3.6)	1 (0.9)	5 (3.1)	5 (3.8)	7 (4.2)	3 (1.9)	20 (3.4)	11 (2.1)
Asthma symptom score (ASS ≥ 2)	14 (10.4)	24 (17.7)	11 (10.0)	21 (19.3)	5 (3.7)	28 (21.4)	8 (4.9)	23 (14.5)	**38 (7.0)**	**96 (17.9)**

Bold figures indicate statistical significance between baseline and follow-up (*p < 0.05*).

### Lung Function Indices

Lung function indices are presented in Supplementary Table S1. Mean FEV_1_ (range 1.5–1.6 L at baseline and 1.7–1.8 L at follow-up) and FVC (range 1.8–1.9 L at baseline and 2.0–2.2 L at follow-up) did not differ by > 10% by area at both baseline and follow-up. The prevalence of airway obstruction (FEV_1_ < LLN) was higher in the urban low-industrialised (23.3%) compared to other areas (<19%) at baseline and in the urban industrialised (30.4%) compared to other areas at follow-up (<27.2%). The prevalence of restrictive lung pattern (FVC < LLN) was highest in the urban industrialised area at both baseline (14.8%) and follow-up (16.8%) compared to other areas. Learners residing in the urban industrialised area had the highest prevalence of airway inflammation (FeNO < 35 ppb) at both baseline (16%) and follow-up (22.2%). As expected (due to 1 year of growth), the overall mean FEV_1_ (1.8 L ± 0.34 vs. 1.6 L ± 0.28), FVC (2.1 L ± 0.39 vs. 1.8 L ± 0.33) and (forced mid-expiratory flow), FEF_25-75_ (2.1 L ± 0.87 vs. 1.9 L ± 0.63) were significantly higher at follow-up compared to baseline. Similarly, the prevalence of airway obstruction as defined by FEV_1_ < LLN (22.4% vs. 17.6%), FEF_25–75_ <LLN (23.3% vs. 14.9%), and FEV_1_/FVC < 0.8 (24.1% vs. 19.1%) were also significantly higher at follow-up compared to baseline.

### New Onset Cases of Asthma-Associated Outcomes at 12 Months Follow Up

While children residing in the low-industrilised area had the highest proportions of new cases of parental-reported ocular-nasal symptoms and rhinitis, the proportions of new cases parental-reported wheezing, ASS and doctor-diagnosed asthma, were the highest in children residing in the peri-urban area (Table 3). Children residing in the urban industrialised area had the highest proportions of new cases of large airway obstruction (FEV_1_ < LLN), small airway obstruction (FEF_25–75_ < LLN), airway obstruction (FEV_1_/FVC < 0.8), and airway inflammation (FeNO > 35 ppb). Overall, ocular-nasal symptoms (31.1%), ASS ≥ 2 (17.8%), airway obstruction (16.4%), small airway obstruction (16.4%) and large airway obstruction (14.5%) were the highest proportions of new cases of asthma-associated outcomes at 12 months follow-up.

**TABLE 3:** New cases of asthma-associated outcomes at 12 months follow-up among learners residing in four informal settlements of the Western Cape (The Association Between Urinary Concentrations of Organophosphate Metabolites and Asthma-Related Outcomes Among Schoolchildren From Informal Settlements, Western Cape, South Africa. 2015–2016).

	*N*	Marconi-beam	Masiphumelele	Khayelitsha	Oudtshoorn	All areas
		Urban industrialised	Urban low-industrialised	Peri-urban	Rural	
Ocular-nasal symptoms	405	37 (33)	43 (57.3)	34 (33.7)	12 (10.3)	126 (31.1)
Rhinitis	418	2 (2.04)	7 (10.5)	8 (6.7)	0 (0)	17 (4.1)
Wheezing	456	8 (6.7)	5 (6.3)	9 (7.6)	1 (0.7)	23 (5.04)
Doctor diagnosed asthma	509	2 (1.5)	1 (1.01)	5 (3.9)	3 (1.9)	11 (2.2)
Asthma symptom score ≥ 2	455	16 (14.6)	19 (20.8)	26 (23.4)	20 (13.9)	81 (17.8)
FEV_1_ < LLN	382	21 (21.7)	10 (14.3)	14 (14.9)	14 (11.6)	59 (14.5)
FVC < LLN	408	12 (12)	7 (8.5)	7 (6.9)	12 (9.7)	38 (9.3)
FEV_1_/FVC < 0.8	395	24 (24.5)	12 (16.4)	12 (12)	17 (13.7)	65 (16.5)
FEF _25–75_ <LLN	378	23 (25)	10 (14.5)	13 (13.5)	16 (13.2)	62 (16.4)
Airway inflammation (FeNO > 35 ppb)	520	12 (10.8)	5 (5.8)	9 (7.1)	2 (1.4)	28 (5.9)

New cases calculated as proportion of cases among case-free participants at baseline.

### Median Urinary DAP Concentrations

At baseline, five children (0.8%) had DEP concentrations below LOQ, while only two children (0.3%) had DMP concentration below LOQ (Table 4). At follow-up 161 children (29.9%) had DEP concentrations below LOQ, and 33 children (6.1%) had DMP concentrations below LOQ.

**TABLE 4:** Distribution of DAP metabolites (ng/mL) across the four study areas at the baseline (2015) and follow-up (2016) studies (The Association Between Urinary Concentrations of Organophosphate Metabolites and Asthma-Related Outcomes Among Schoolchildren From Informal Settlements, Western Cape, South Africa. 2015–2016).

DAPs (ng/mL)	Baseline					Follow up				
	Marconi-beam urban industrialised	Masiphumelele urban low-industrialised	Khayelitsha peri-urban	Oudtshoorn rural	Total	Marconi-beam urban industrialised	Masiphumelele urban low-industrialised	Khayelitsha peri-urban	Oudtshoorn	Total
	*N* = 150	*N* = 117	*N* = 152	*N* = 170	*N* = 589	*N* = 126	*N* = 97	*N* = 124	*N* = 145	*N* = 492
DEP	1.4 (0.9–2.8)	1.9 (1.5–3.5)	3.7 (2.1–6.2)	2.6 (1.4–4.5)	2.3 (1.3–4.3)	2.2 (1.1–4.4)	2.3 (1.1–3.6)	2.4 (1.1–4.2)	2.8 (1.7–5.7)	2.5 (1.1–4.3)
DMP	13.2 (7.5–20.1)	14.3 (7.3–26.3)	14.8 (7.7–27.6)	12.6 (6.3–19.9)	13.7 (7.3–22.2)	15.4 (6.5–35.5)	13 (4.7–21.9)	13.1 (7.6–23.1)	21.2 (10.2–34.2)	15.6 (8–28.6)
DMTP	10.9 (5.6–17.6)	16.7 (8.8–28)	13.7 (7–26.2)	13.7 (6.7–26.7)	13 (6.9–23.8)					
∑DAP*	26.9 (16.7–41.9)	37.9 (24.2–55.4)	34.8 (19.5–63.1)	34.3 (17.5–53.7)	32.9 (18.4–52.9)	19.4 (8.4–38.2)	16.4 (8.3–25.5)	15.6 (9.5–27.9)	24.8 (14.1–41)	19.3 (10.4–34.1)
Average DAPs over cohort period										
Average DEP	NA	NA	NA	NA	NA	2.3 (1.4–3.7)	2.3 (1.4–3.7)	3.3 (2.2–4.8)	2.9 (2.1–5)	2.8 (1.8–4.4)
Average DMP	NA	NA	NA	NA	NA	17.2 (10.2–30.2)	14.8 (9.7–24.6)	15.1 (9.3–24.3)	17.9 (11.6–27.6)	17 (10.2–27.2)
Ave DEP DMP	NA	NA	NA	NA	NA	20.9 (12.6–32.2)	17.9 (11.9–30.3)	20.5 (12.1–29.3)	22.1 (14.5–32.2)	20.9 (12.9–31.9)
Ave DAP	NA	NA	NA	NA	NA	32.6 (22.5–53.7)	39.6 (25.8–58.5)	34.1 (21.9–55.8)	40.4 (26.9–57.9)	37.8 (23.4–56.7)

Data presented as median (IQR), measured in ng/mL. DMP, dimethyl phosphate; DMTP, dimethyl thiophosphate; DEP, diethyl phosphate; NA, Not available.

∑DAP* = sum DAPs at baseline (DEP + DMP + DMTP) and DAP at follow-up = (DEP + DMP); DMTP not measured at follow-up.

Average DEP: (baseline DEP + follow-up DEP)/2; Average DMP: (baseline DMP + follow-up DMP)/2.

Ave DEP DMP = average DEP + average DMP.

Ave DAP = average DEP + average DEP + baseline DMTP.

Values below LOQ were substituted by LOQ divided by square root of 2.

The median DEP and DMP concentrations for all areas (Total in Table 4) at baseline were not substantially different to those at follow-up (DMTP not measured at follow-up). Learners residing in the low industrialised area had the highest median concentration of ∑DAP (37.9 ng/mL, IQR: 24.2–55.4) at baseline (sum of DEP, DMP and DMTP) compared to the other three study areas, while those in the rural area had the highest median ∑DAP (24.8 ng/mL, IQR: 14.1–41) concentrations at follow-up (sum of DEP and DMP).

### Associations Between DAP Metabolites and Asthma Associated Outcomes

There were no significant associations or consistent patterns of increased risk of new asthma-associated outcomes during the 12 months period apart from a consistent non-statistically significant, positive association between average DAP concentrations and airway inflammation (Table 5).

**TABLE 5:** Associations between new cases of asthma-associated outcomes and average OP pesticides (ng/mL) exposure over the 12 months period in multivariate models (The Association Between Urinary Concentrations of Organophosphate Metabolites and Asthma-Related Outcomes Among Schoolchildren From Informal Settlements, Western Cape, South Africa. 2015–2016).

	Number of new cases N (%)	Average DEP	Average DMP	Average DEP DMP	Average DAP
Ocular-nasal symptoms	126 (31.1)	0.84 (0.56, 1.25)	1.03 (0.72, 1.47)	0.91 (0.61, 1.37)	0.42 (0.47, 1.11)
Rhinitis	17 (4.1)	0.96 (0.34, 2.69)	1.17 (0.53, 2.58)	1.24 (0.47, 3.27)	1.65 (0.54, 5.07)
Wheezing	23 (5.0)	0.43 (0.18, 1.05)	0.55 (0.28, 1.11)	0.41 (0.21, 1.03)	0.49 (0.20, 1.16)
Doctor diagnosed asthma	11 (2.2)	0.65 (0.13, 3.27)	1.17 (0.36, 3.74)	0.68 (0.12, 3.84)	0.62 (0.09, 4.05)
Asthma symptom score	81 (17.8)	0.62 (0.39, 1.00)	0.77 (0.53, 1.13)	0.65 (0.42, 1.01)	0.73 (0.45, 1.18)
FEV_1_ < LLN^a^	59 (15.5)	0.65 (0.39, 1.09)	0.87 (0.58, 1.29)	0.80 (0.51, 1.28)	0.85 (0.52, 1.39)
FVC < LLN^a^	38 (9.3)	0.72 (0.42, 1.32)	0.82 (0.53, 1.28)	0.71 (0.42, 1.19)	0.76 (0.44, 1.31)
FEV_1_/FVC < 0.8	65 (16.5)	0.73 (0.44, 1.23)	1.08 (0.71, 1.64)	1.20 (0.73, 1.99)	1.01 (0.60, 1.70)
FEF _25–75_ < LLN^a^	62 (16.4)	0.62 (0.36, 1.05)	0.79 (0.52, 1.20)	0.72 (0.44, 1.18)	0.65 (0.39, 1.09)
Airway inflammation (FeNO > 35 ppb)	28 (5.9)	1.81 (0.88, 3.74)	1.24 (0.62, 2.45)	1.22 (0.56, 2.61)	1.45 (0.67, 3.16)

Data presented as OR (95% CI). Adjusted for age, sex, BMI, maternal smoking, atopy, presence of smokers in the house, use of paraffin for cooking, annual NO_2_ pollutant, annual PM_2.5_ pollutant and study area.

Average DEP: (baseline DEP + follow-up DEP)/2; Average DMP: (baseline DMP + follow-up DMP)/2; Ave DEP DMP = average DEP + average DMP; Ave DAP = average.

^a^ Not adjusted-for age and sex and BMI for these variables.

Effect estimated reflects change per ng/mL increase in DAP metabolite concentrations.

New cases of asthma-like outcomes based on proportion with the outcome of interest at 12 months follow-up among children case-free at baseline.

Table 6 summarizes the cross-sectional association between DAP concentrations and asthma-outcomes using a linear mixed-effect model to account for repeated measurements at the individual level (random effects). Increasing concentrations of DEP were consistently mostly non-significantly associated with a decrease in FEV_1_, with no consistent dose-dependent relationship. There were no consistent relationships between DEP concentrations and FVC and FEV_1_/FVC. However, compared to the lowest DEP quintile category (<1.1 ng/mL), schoolchildren at the third quintile (1.89–3.01 ng/mL) have a significantly increased FeNO levels in the fully-adjusted model (*β* = 2.99; 95% CI: 0.48, 5.50).

**TABLE 6:** Summary of the repeated cross-sectional analysis of DAP concentrations and asthma-related outcomes at the baseline (2015) and follow-up (2016) studies using a random and fixed effects model (The Association Between Urinary Concentrations of Organophosphate Metabolites and Asthma-Related Outcomes Among Schoolchildren From Informal Settlements, Western Cape, South Africa. 2015–2016).

		Quintile 1 (ref)	Quintile 2	Quintile 3	Quintile 4	Quintile 5
DEP (ng/mL)		1.1	1.10–1.88	1.89–3.01	3.03–5.02	5.03–148
FEV_1_ (litres)	Unadjusted	0.00	**−0.097 (−0.147, −0.046)**	−0.027 (−0.074, 0.019)	**−0.054 (−0.102, −0.006)**	−0.042 (−0.089, 0.006)
	Adjusted, basic		−0.019 (−0.063, 0.026)	−0.011 (−0.051, 0.028)	−0.017 (−0.059, 0.024)	−0.008 (−0.049, 0.032)
	Adjusted, extended		−0.012 (−0.058, 0.034)	−0.018 (−0.060, 0.024)	−0.020 (−0.064, 0.024)	−0.014 (−0.057, 0.029)
FVC (litres)	Unadjusted	0.00	**−0.159 (−0.221, −0.096)**	0.045 (−0.103, 0.012)	**−0.073 (−0.132, −0.015)**	−0.059 (−0.118, −0.001)
	Adjusted, basic		−0.047 (−0.956, 0.002)	−0.005 (−0.049, 0.038)	0.004 (−0.413, 0.049)	0.003 (−0.042, 0.478)
	Adjusted, extended		−0.045 (−0.095, 0.005)	−0.012 (−0.058, 0.034)	0.004 (−0.044, 0.052)	−0.003 (−0.051, 0.044)
FEV_1_/FVC	Unadjusted	0.00	**−26.64 (−34.82, −18.45)**	−4.39 (−11.75, 2.97)	−7.03 (−14.58, 0.51)	−5.52 (−12.95, 1.92)
	Adjusted, basic		0.68 (−1.21, 2.57)	0.62 (−1.07, 2.31)	−0.33 (−2.09, 1.42)	0.81 (−0.93, 2.55)
	Adjusted, extended		0.79 (−1.15, 2.73)	0.54 (−1.23, 2.31)	−0.77 (−2.62, 1.08)	0.76 (−1.06, 2.59)
Average FeNO (ppb)	Unadjusted	0.00	−1.79 (−4.27, 0.67)	1.51 (−0.84, 3.86)	−2.02 (−4.34, 0.29)	1.14 (−1.19, 3.48)
	Adjusted, basic		−0.83 (−3.46, 1.81)	**2.69 (0.28, 5.11)**	−1.37 (−3.83, 1.08)	1.35 (−1.09, 3.78)
	Adjusted, extended		−0.89 (−3.57, 1.79)	**2.99 (0.48, 5.50)**	−0.79 (−3.37, 1.77)	1.18 (−1.36, 3.71)
DMP (ng/mL)		1.1–5.9	6.0–11.3	11.4–17.8	17.9–29.8	30.0–227
FEV_1_ (litres)	Unadjusted	0.00	−0.034 (−0.084, 0.015)	−0.011 (−0.061, 0.039)	0.008 (−0.044, 0.059)	0.020 (−0.031, 0.071)
	Adjusted, basic		−0.001 (−0.043, 0.041)	0.021 (−0.021, 0.063)	0.031 (−0.012, 0.075)	0.011 (−0.033, 0.054)
	Adjusted, extended		0.002 (−0.041, 0.047)	0.019 (−0.023, 0.063)	0.026 (−0.019, 0.071)	0.004 (−0.042, 0.049)
FVC (litres)	Unadjusted	0.00	−0.039 (−0.101, 0.022)	0.032 (−0.095, 0.029)	−0.011 (−0.074, 0.053)	0.017 (−0.046, 0.080)
	Adjusted, basic		0.009 (−0.036, 0.056)	0.008 (−0.038, 0.054)	0.027 (−0.021, 0.075)	0.023 (−0.025, 0.072)
	Adjusted, extended		0.012 (−0.036, 0.061)	0.002 (−0.046, 0.050)	0.018 (−0.031, 0.068)	0.016 (−0.034, 0.067)
FEV_1_/FVC	Unadjusted	0.00	−5.36 (−13.34, 2.61)	−2.28 (−10.33, 5.76)	2.33 (−5.71, 10.37)	8.25 (0.22, 16.29)
	Adjusted, basic		0.41 (−1.38, 2.21)	0.26 (−1.38, 2.21)	1.11 (0.77, 2.99)	0.93 (−0.96, 2.82)
	Adjusted, extended		0.53–1.34, 2.40)	0.29 (−1.54, 2.14)	0.98 (−0.97, 2.92)	0.74 (−1.23, 2.72)
Average FeNO	Unadjusted	0.00	0.73 (−1.71, 3.17)	−0.48 (−2.92, 1.96)	0.38 (−2.14, 2.91)	1.95 (−0.542, 4.44)
	Adjusted, basic		1.49 (−1.06, 4.04)	0.31 (−2.24, 2.46)	−0.14 (−2.74, 2.46)	1.80 (0.83, 4.44)
	Adjusted, extended		1.57 (−1.04, 4.18)	0.13 (−2.45, 2.72)	0.10 (−2.54, 2.74)	2.36 (−0.34, 5.06)
DAP (ng/mL)		2.2–12.0	12.1–20.4	20.5–31.4	31.5–51.7	51.8–233.5
FEV_1_ (litres)	Unadjusted	0.00	−0.016 (−0.065, 0.032)	**−0.054 (−0.102, −0.070)**	**−0.097 (−0.145, -0.048)**	**−0.097 (−0.145, −0.048)**
	Adjusted, basic		0.039 (−0.002, 0.081)	0.021 (−0.020, 0.063)	0.004 (−0.039, 0.047)	0.015 (−0.027, 0.059)
	Adjusted, extended		0.041 (−0.003, 0.085)	0.020 (−0.024, 0.064)	−0.002 (−0.047, 0.043)	0.006 (−0.039, 0.052)
FVC (litres)	Unadjusted	0.00	**−0.069 (−0.129, −0.009)**	**−0.087 (−0.148, −0.028)**	**−0.129 (−0.189, −0.069)**	**−0.176 (−0.236, −0.117)**
	Adjusted, basic		0.006 (−0.041, 0.052)	0.014 (−0.032, 0.060)	0.011 (−0.036, 0.059)	−0.013 (−0.061, 0.035)
	Adjusted, extended		0.003 (−0.045, 0.052)	0.008 (−0.041, 0.057)	−0.001 (−0.041, 0.057)	−0.026 (−0.077, 0.024)
FEV_1_/FVC	Unadjusted	0.00	**−10.98 (−18.67, −3.29)**	**−18.39 (−26.04, −10.75)**	**−27.72 (−35.42, −20.02)**	**−31.78 (−39.50, −24.06)**
	Adjusted, basic		1.27 (−0.49, 3.04)	−0.64 (−2.41, 1.13)	−0.46 (−2.32, 1.39)	−0.085 (−1.94, 1.77)
	Adjusted, extended		1.43 (−0.42, 3.28)	−0.66 (−2.52, 1.21)	−0.61 (−2.55, 1.33)	−0.43 (−2.38, 1.53)
Average FeNO	Unadjusted	0.00	−1.08 (−3.55, 1.39)	0.64 (−1.77, 3.04)	−0.23 (−2.67, 2.20)	−0.031 (−2.44, 2.38)
	Adjusted, basic		−0.44 (−3.01, 2.12)	0.84 (−1.70, 3.38)	0.29 (−2.33, 2.93)	0.75 (−1.88, 3.38)
	Adjusted, extended		−0.38 (−3.04, 2.28)	0.72 (−1.89, 3.34)	0.22 (−2.46, 2.92)	0.89 (−1.84, 3.62)

Basic model: age, sex BMI, child birth weight and Atopy. Extended model: age, sex BMI, child birth weight, atopy cooking with paraffin, maternal smoking, smokers in the house, annual NO_2_ and annual PM_2.5_, and study area. Bolded values indicate a statistically significant association (*p* < 0.05).

There was an association between PEF and sum DAP (*β* = −0.093; 95% CI: −0.186, −0.001) (Supplementary Table S4) and between FVC < LLN and DAP metabolites: DMP (Odds Ratio (OR) = 1.39, 95% confidence interval (CI): 1.01, 1.91) and DMPT (OR = 1.77; 95% CI: 1.22, 2.42) at baseline (Supplementary Table S5).

In girls, DEP, DMP, and DMTP and sum DAP were significantly associated with the increased odds of FVC < LLN at baseline (Supplementary Table S6a). However, this response was reversed at follow-up with DMP and sum DAP significantly associated with the decreased odds of FVC < LLN. Female learners also demonstrated an increased risk of airway inflammation (FeNO > 35 ppb) with DMP (OR = 1.82; 95% CI: 1.17, 2.84) and sum DAP (OR = 1.81; 95% CI 1.11, 2.93) at follow-up (Supplementary Table S6a), while male learners demonstrated an increased risk of elevated inflammation (FeNO > 15 ppb) with DMTP (OR = 1.36; 95% CI: 1.02, 1.81) and sum DAP (OR: 1.42; 95% CI: 1.01, 1.92) at follow-up. No evidence of effect modification was observed by atopy (Supplementary Tables S7, S8).

## Discussion

This study investigated the association between non-agricultural OP pesticide exposures measured through urinary DAPs and asthma-related outcomes in learners residing in four informal settlements in a South African setting, which to our knowledge, had not previously been reported.

The median urinary ∑DAP concentrations measured at the baseline study were higher (32.9 ng/mL ≈ 82 nmol/L) than that observed in schoolchildren living in urban and agricultural communities of Spain [21]. DMTP concentrations in the current study also were higher (13 ng/mL) than those of children in an US urban community (6.5 ng/mL) [22]. Learners residing in SA’s agricultural intensive areas had been found to have higher urinary DAP concentrations [median = 68.3 (27.9–129.5)] compared to our study population [23]. In non-agricultural communities of SA, high residential pesticide exposure can occur in lower socioeconomic settings. In this current study, there was a fairly comparable distribution of urinary DEP and DMP metabolite levels at both the baseline and follow-up studies of learners, suggesting consistency in OP pesticides exposure throughout the 12 months period (Table 4 and Supplementary Figure S1).

The reason for no consistent associations between urinary DAP concentrations and new cases of asthma-related outcomes at 12 months follow-up from baseline found could be that the follow-up period of 12 months was too short. Furthermore, there were only two repeated samples of urinary DAPs collected over the 12 months study period, which may not represent OP exposure over the period, since pesticides are eliminated from the body within 24 h and may vary seasonally.

In the cross-sectional analysis some associations were observed between total DAP and reduced peak expiratory flow, as well as between DAP metabolites and reduced FVC (FVC < LLN) at baseline. Previous studies conducted in children in agricultural settings have shown associations between urinary DAP metabolites and asthma-associated outcomes including wheezing, cough, shortness of breath, reduced lung function and asthma [7, 24–26]. However, most of these studies were birth cohorts, which followed children from the prenatal period up to the age of 7 years post-natally. In two of these studies, urinary DAP metabolites were measured twice at prenatal phase (in pregnant mothers) and five times after birth from age 0.5, 1, 2, 3 and 5 years [7, 26]. A non-birth cohort study also showed an association between urinary DAP metabolites and asthma exacerbation through increased Th2 cytokine production in children aged 6–16 years at 4 months follow-up [24]. Evidence from non-agricultural settings, however, is limited and inconsistent. A cross-sectional survey of school-aged children in the general population of the US reported no association between DAP metabolites and respiratory symptoms [15] while a case-control study conducted in the US reported an increased asthma risk with pesticide exposure: herbicides (OR = 4.58; 95% CI: 1.36, 15.43), pesticides (OR = 2.39; 95% CI: 1.17, 4.89) [4]. Two studies in the US [12, 13] and one in Lebanon [14] found an increased odds of asthma, wheezing and dry cough among children with increased pesticide use in homes. However, in the latter three studies, pesticide exposure was assessed through interviews and the type of pesticides were not specified. Reardon et.al [12]. also found an association between pre-natal OP and pyretroid metabolite air concentrations and respiratory symptoms and IgE production among 5 years-old children.

A previous study in the rural areas of the Western Cape found that levels of DAP and 3,5,6-trichloro-2-pyridinol (TCPY), the chlorpyrifos specific metabolite, were associated with airway inflammation and increased serum levels of inflammatory cytokines in rural farm and non-farm women workers [9]. In the current study, there was some evidence of an association between DEP metabolite and an increase in FeNO (*β* = 2.99; 95% CI: 0.48–5.50). A relationship between airway inflammation and DAP metabolite is plausible since OP-mediated acetylcholinesterase (AChE) inhibition may occur following acute or chronic exposures, resulting in bronchoconstriction and airway inflammation [27, 28].

Sex differences in lung function and pathophysiology of respiratory disease including asthma related-outcomes have been well documented regardless of age [29–32]. In sensitivity analysis, there was some evidence of small airway obstruction (FEF_25–75_ < LLN) at baseline and airway inflammation (FeNO > 35 ppb) at follow-up associated with DAP metabolites in female learners.

There were some limitations in the study. Only three DAP metabolites out of six metabolites were measured, however, they do cover the full spectrum of DAP producing OP pesticides used in South Africa [23]. Additionally, the use of single non-first morning void urine samples and lack of correction for hydration through creatinine adjustment are further limitations that may have led to underestimation and intra and inter-individual variability in urinary DAP concentrations [33] that could have biased associations towards the null. The lack of data on reported pesticide usage, other environmental pesticide exposures and parental pesticide exposures in this study precluded us from investigating the association between these exposure variables and respiratory outcomes found in previous studies [4, 13–15]. The large unexplained improvement in the prevalence of rhinitis and wheezing at follow-probably reduced the effect size of pesticide exposure on these outcomes.

### Conclusion

This study did not find a consistent association between residential OP pesticide exposure and new cases of asthma-related outcomes in schoolchildren residing in four informal settlements despite the measured levels being higher than other non-agricultural settings. However, the relevance of these findings needs to be explored further in a larger study with a longer follow-up period.
