# Supplementary material for: The Association Between Urinary Concentrations of Organophosphate Metabolites and Asthma-Related Outcomes Among Schoolchildren From Informal Settlements
Source: Int J Public Health. 2023 Aug 22;68:1606174. doi: 10.3389/ijph.2023.1606174 (PMC10477361; doi:10.3389/ijph.2023.1606174)
Supplement: Supplementary file 1 [file DataSheet1.docx]

**Supplementary Information**

Table S1: Lung function indices among learners living in the informal settlements of the Western Cape at the baseline (2015) and follow-up (2016) studies.

|  | **Marconi Beam**  Urban industrialized | | **Masiphumelele**  Urban low-industrialized | | **Khayelitsha**  Peri-urban | | **Oudtshoorn**  Rural | | **All areas** | |
| --- | --- | --- | --- | --- | --- | --- | --- | --- | --- | --- |
|  | **Baseline**  **N = 150** | **Follow-up**  **N = 136** | **Baseline**  **N = 117** | **Follow-up**  **N = 109** | **Baseline**  **N = 163** | **Follow-up**  **N = 131** | **Baseline**  **N = 170** | **Follow-up**  **N = 159** | **Baseline**  **N = 600** | **Follow-up**  **N =535** |
| FEV_1_, litres | 1.6 ± 0.29 | 1.8 ± 0.39 | 1.6 ± 0.31 | 1.8 ± 0.35 | 1.5 ± 0.23 | 1.7 ± 0.29 | 1.5 ± 0.27 | 1.7 ± 0.34 | **1.6 ± 0.28** | **1.8 ± 0.34** |
| FVC, litres | 1.9 ± 0.33 | 2.1 ± 0.44 | 1.9 ± 0.34 | 2.2 ± 0.41 | 1.8 ± 0.29 | 2.0 ± 0.33 | 1.8 ± 0.32 | 2.0 ± 0.38 | **1.8 ± 0.33** | **2.1 ± 0.39** |
| FEF _25-75_, litres | 2.0 ± 0.63 | 2.0 ± 0.77 | 2.0 ± 0.72 | 2.0 ± 0.78 | 1.8 ± 0.59 | 2.1 ± 1.21 | 2.0 ± 0.57 | 2.1 ± 0.68 | **1.9 ± 0.63** | **2.1 ± 0.87** |
| FEV_1_ < LLN | 24 (17.8) | 38 (30.4) | 24 (23.3) | 26 (27.1) | 24 (18.6) | 23 (18.9) | 19 (12.6) | 26 (18.1) | **91 (17.6)** | **119 (22.4)** |
| FVC < LLN | 20 (14.8) | 21 (16.8) | 12 (11.7) | 14 (14.6) | 16 (12.4) | 13 (10.7) | 16 (10.6) | 21 (14.6) | 64 (12.4) | 75 (14.1) |
| FEV_1_/FVC < 0.8 | 23 (17.0) | 42 (33.6) | 19 (18.5) | 27 (28.1) | 19 (14.7) | 21 (17.2) | 16 (10.6) | 24 (16.7) | **77 (14.9)** | **124 (23.3)** |
| FEF _25-75_ < LLN | 32 (23.7) | 46 (36.8) | 25 (24.3) | 28 (29.2) | 23 (17.7) | 21 (17.2) | 19 (12.6) | 24 (16.7) | **99 (19.1)** | **128 (24.1)** |
| FeNO, ppb * | 13 (9 – 25) | 15 (10 – 32) | 15 (11 – 25) | 11 (8 – 20) | 14 (10 –18) | 13 (9 – 20) | 10 (8 – 15) | 11 (8 – 17) | 13 (9 – 19) | 12 (9 – 20) |
| FeNO > 15 ppb | 65 (43.3) | 63 (50.0) | 60 (51.3) | 37 (38.1) | 63 (41.2) | 49 (40.8) | 41 (24.3) | 50 (35.2) | 229 (38.9) | 219 (41.2) |
| FeNO > 35 ppb | 24 (16) | 28 (22.2) | 17 (14.5) | 14 (4.4) | 13 (8.5) | 15 (12.5) | 4 (2.4) | 5 (3.5) | 58 (9.9) | 69 (12.9) |

Continuous data presented as mean ± SD, categorical data as N (%), * median (IQR) due to skewed distribution.

Bold figures indicate statistical significance between baseline and follow-up (p < 0.05).

FEF_25-75_: forced mid-expiratory flow; FeNO: fractional exhaled nitric oxide; FEV_1_: forced expiratory volume in 1 second; FVC: forced vital capacity; LLN: lower limit of normal below the 5^th^ percentile.

**Table S2: Linear regression showing bivariate association between pesticides metabolites and lung function indices at baseline and follow-up**

|  | **Baseline** | | | | **Follow-up** | | |
| --- | --- | --- | --- | --- | --- | --- | --- |
|  | DEP | DMP | DMTP | ∑DAP | DEP | DMP | ∑DAP |
| FEV_1_ (litres) | **-0.030 (-0.05, -0.006)** | -0.004 (-0.028, 0.019) | -0.005 (-0.03, 0.021) | -0.008 (-0.037, 0.022) | 0.005 (-0.031, 0.043) | -0.002 (-0.028, 0.247) | 0.002 (-0.029, 0.033) |
| FVC  (litres) | **-0.034 (-0.061, -0.006)** | -0.008 (-0.036, 0.019) | -0.011 (-0.041, 0.018) | -0.015 (-0.049, 0.018) | -0.013 (-0.055, 0.029) | -0.013 (-0.044, 0.016) | -0.011 (0.046, 0.024) |
| PEF  (litres) | **-0.106 (-0.175, -0.036)** | -0.043 (-0.112, 0.267) | -0.037 (-0.112, 0.037) | -0.064 (-0.150, 0.021) | 0.049 (-0.062, 0.162) | 0.015 (-0.065, 0.097) | 0.029 (-0.065, 0.122) |
| FEF _25-75_  (litres) | -0.039 (-0.092, 0.014) | -0.006 (-0.058, 0.046) | 0.015 (-0,041, 0.071) | 0.0001 (-0.065, 0.065) | 0.026 (-0.066, 0.120) | 0.006 (-0.061, 0.074) | 0.009 (-0.068, 0.088) |
| FeNO  (ppb) | 0.019 (-0.034, 0.072) | 0.028 (-0.024, 0.080) | 0.019 (-0.036, 0.075) | 0.039 (-0.025, 0.104) | -0.008 (-0.084, 0.069) | 0.031 (-0.023, 0.086) | 0.027 (-0.036, 0.092) |

Data presented as Beta coefficient (95 % CI), bolded figures indicate a statistically significant association
DMP: dimethyl phosphate, DMTP: dimethyl thiophosphate, DEP: diethyl phosphate
FEF_25-75_: forced mid-expiratory flow; FeNO: fractional exhaled nitric oxide; FEV_1_: forced expiratory volume in 1 second; FVC: forced vital capacity.Effect estimated reflects change per ng/ml increase in DAP metabolite concentrations.

**Table S3: Logistic regression showing bivariate association between pesticides metabolites and lung function indices at baseline and follow-up**

|  | **Baseline** | | | **Follow-up** | |
| --- | --- | --- | --- | --- | --- |
|  | DEP | DMP | DMPT | DEP | DMP |
| FEV_1_ < LLN | 1.13 (0.89, 1.42) | 1.06 (0.85, 1.33) | 1.11 (0.87, 1.41) | 0.87 (0.67, 1.13) | 0.86 (0.71, 1.02) |
| FVC < LLN | 1.08 (0.83, 1.34) | **1.38 (1.04, 1.83)** | **1.55 (1.15, 2.08)** | 1.02 (0.75, 1.38) | 0.89 (0.72, 1.11) |
| FEV_1_ / FVC < 0.8 | 1.09 (0.86, 1.39) | 0.99 (0.79, 1.26) | 0.92 (0.71, 1.18) | 0.81 (0.63, 1.05) | 0.91 (0.76, 1.09) |
| FEF _25-75_ < LLN | 1.07 (0.86, 1.33) | 1.00 (0.81, 1.24) | 0.97 (0.78, 1.23) | 0.83 (0.64, 1.07) | 0.89 (0.74, 1.06) |
| FENO > 15 ppb | 1.09 (0.92, 1.20) | 1.10 (0.94, 1.31) | 1.12 (0.94, 1.34) | 0.90 (0.73, 1.13) | 1.08 (0.93, 1.27) |
| FENO > 35 ppb | 0.94 (0.72, 1.22) | 0.89 (0.69, 1.15) | 0.96 (0.73, 1.28) | 1.11 (0.81, 1.51) | 1.08 (0.85, 1.36) |

Data presented as OR (95% CI), bold figures indicate a statistically significant association
DMP: dimethyl phosphate, DMTP: dimethyl thiophosphate, DEP: diethyl phosphate
FEF_25-75_: forced mid-expiratory flow; FeNO: fractional exhaled nitric oxide; FEV_1_: forced expiratory volume in 1 second; FVC: forced vital capacity; LLN: lower limit of normal below the 5^th^ percentile.

Effect estimated reflects change per ng/ml increase in DAP metabolite concentrations.

**Table S4: Linear regression models showing adjusted associations between pesticide metabolites and lung function indices at baseline and 12-months follow-up**

|  | **Baseline** | | | | **Follow-up** | | |
| --- | --- | --- | --- | --- | --- | --- | --- |
|  | DEP | DMP | DMTP | ∑DAP | DEP | DMP | ∑DAP |
| FEV_1_ | -0.025 (-0.051, 0.001) | -0.012 (-0.037, 0.013) | -0.009 (-0.036, 0.0178 | -0.017 (-0.048, 0.013) | 0.012 (-0.027, 0.052) | 0.012 (-0.017, 0.041) | 0.015 (-0.018, 0.049) |
| FVC | -0.021 (-0.049, 0.007) | -0.018 (-0.045, 0.009) | -0.023 (-0.053, 0.006) | -0.032 (-0.066, 0.002) | 0.003 (-0.039, 0.046) | 0.012 (-0.019, 0.044) | 0.015 (-0.021, 0.052) |
| PEF | -0.088 (-0.167, 0.011) | -0.060 (-0.134, 0.014) | -0.060 (-0.141, 0.021) | **-0.093 (-0.186, -0.001)** | 0.063 (-0.059, 0.184) | 0.051 (-0.038, 0.140) | 0.061 (-0.042, 0.164) |
| FEF_25-75_ | -0.044 (-0.106, 0.018) | -0.009 (-0.068, 0.049) | 0.025 (-0.034, 0.090) | -0.002 (-0.075, 0.072) | 0.058 (-0.028, 0.145) | 0.128 (-0.050, 0.076) | 0.020 (-0.053, 0.093) |
| FeNO | 0.037 (-0.017, 0.092) | 0.012 (-0.039, 0.063) | -0.004 (-0.059, 0.051) | 0.016 (-0.049, 0.080) | -0.003 (-0.0790, 0.073) | 0.021 (-0.035, 0.076) | 0.016 (-0.048 .080) |

Data presented as Beta coefficient (95 % CI)

Adjusted for age, sex, BMI, low birth weight, atopy, maternal smoking, presence of smokers in the house, use of paraffin for cooking, annual NO_2_ pollutant, annual PM_2.5_ pollutant and study area.

Effect estimated reflects change per ng/ml increase in DAP metabolite concentrations.

**Table S5: Logistic regression models showing adjusted associations between pesticide metabolites and lung function indices at baseline and 12-months follow-up**

|  | **Baseline** | | | | **Follow-up** | | |
| --- | --- | --- | --- | --- | --- | --- | --- |
|  | DEP | DMP | DMPT | ∑DAP | DEP | DMP | ∑DAP |
| FEV_1_ < LLN* | 1.25 (0.95, 1.65) | 1.07 (0.83, 1.34) | 1.15 (0.88, 1.51) | 1.09 (0.80, 1.45) | 0.88 (0.66, 1.18) | 0.88 (0.71, 1.08) | 0.89 (0.71, 1.12) |
| FVC < LLN* | 1.18 (0.85, 1.62) | **1.39 (1.01, 1.91)** | **1.77 (1.22, 2.42)** | **1.64 (1.13, 2.37)** | 0.97 (0.60, 1.36) | 0.89 (0.69, 1.13) | 0.89 (0.69, 1.18) |
| FEV_1_ / FVC < 0.8 | 1.14 (0.85, 1.52) | 0.91 (0.71, 1.18) | 0.83 (0.62, 1.15) | 0.86 (0.63, 1.17) | 0.86 (0.64, 1.14) | 0.99 (0.80, 1.22) | 0.93 (0.75, 1.17) |
| FEF _25-75_ < LLN* | 1.27 (0.97, 1.67) | 1.00 (0.79, 1.26) | 1.06 (0.82, 1.38) | 1.05 (0.79, 1.40) | 0.87 (0.65, 1.16) | 0.93 (0.76, 1.14) | 0.88 (0.70, 1.09) |
| FENO > 15 ppb | 1.19 (0.97, 1.48) | 1.04 (0.85, 1.26) | 0.99 (0.79, 1.24) | 1.15 (0.92, 1.44) | 0.91 (0.69, 1.18) | 1.05 (0.86, 1.27) | 1.07 (0.88, 1.31) |
| FENO > 35 ppb | 1.22 (0.83, 2.17) | 0.94 (0.61, 1.32) | 0.84 (0.56, 1.26) | 0.91 (0.63, 1.32) | 1.26 (0.81, 1.95) | 1.01 (0.72, 1.39) | 1.21 (0.91, 1.62) |

Data presented as OR (95 % CI), bold figures indicate a statistically significant association.

Adjusted for age, sex, BMI, low birth weight, atopy maternal smoking, presence of smokers in the house, use of paraffin for cooking, annual NO_2_ pollutant, annual PM_2.5_ pollutant and study area.

*not adjusted-for age and sex and BMI as these variables have already been accounted-for in the calculation of the lower limits of normal (LLN).

Effect estimated reflects change per ng/ml increase in DAP metabolite concentrations.

**Table S6a:** **Logistic regression models showing adjusted associations between pesticide metabolites and lung function indices in females at baseline and 12 months follow-up.**

|  | **Baseline** | | | | **Follow-up** | | |
| --- | --- | --- | --- | --- | --- | --- | --- |
|  | DEP | DMP | DMPT | ∑DAP | DEP | DMP | ∑DAP |
| FEV_1_ < LLN* | 1.40 (0.96, 2.04) | 1.26 (0.90, 1.75) | 1.25 (0.88, 1.79) | 1.44 (0.94, 2.20) | 0.75 (0.47, 1.19) | 0.78 (0.57, 1.08) | 0.73 (0.50, 1.06) |
| FVC < LLN* | **1.74 (1.05, 2.91)** | **1.99 (1.19, 3.33)** | **1.67 (1.03, 2.66)** | **2.47 (1.33, 4.58)** | 0.85 (0.49, 1.47) | **0.67 (0.47, 0.98)** | **0.63 (0.41, 0.98)** |
| FEV_1_ / FVC < 0.8 | 1.11 (0.70, 1.77) | 1.16 (0.78, 1.72) | 1.08 (0.68, 1.72) | 1.11 (0.66, 1.88) | 0.75 (0.48, 1.18) | 1.09 (0.79, 1.48) | 1.03 (0.72, 1.46) |
| FEF _25-75_ < LLN* | **1.49 (1.03, 2.14)** | 1.29 (0.94, 1.77) | 1.19 (0.85, 1.68) | 1.40 (0.94, 2.08) | 0.68 (0.44, 1.04) | 0.94 (0.70, 1.25) | 0.87 (0.61, 1.20) |
| FENO > 15 ppb | 1.25 (0.94, 1.68) | 0.95 (0.74, 1.21) | 0.83 (0.62, 1.10) | 0.94 (0.68, 1.28) | 0.69 (0.48, 1.02) | 1.28 (0.98, 1.68) | 1.22 (0.89, 1.22) |
| FENO > 35 ppb | 0.87 (0.48, 1.58) | 0.75 (0.49, 1.15) | 0.81 (0.47, 1.39) | 0.72 (0.39, 1.40) | 1.11 (0.65, 1.89) | **1.82 (1.17, 2.84)** | **1.81 (1.11, 2.93)** |

**Table S6b: Logistic regression models showing adjusted associations between pesticide metabolites and lung function indices in males at baseline and 12 months follow-up.**

|  | **Baseline** | | | | **Follow-up** | | |
| --- | --- | --- | --- | --- | --- | --- | --- |
|  | DEP | DMP | DMPT | ∑DAP | DEP | DMP | ∑DAP |
| FEV_1_ < LLN* | 0.98 (0.66, 1.44) | 0.75 (0.52, 1.08) | 1.01 (0.67, 1.52) | 0.79 (0.50, 1.24) | 0.89 (0.62, 1.30) | 0.98 (0.76, 1.26) | 0.99 (0.74, 1.33) |
| FVC < LLN* | 0.82 (0.55, 1.22) | 1.04 (0.70, 1.59) | **1.68 (1.06, 2.66)** | 1.24 (0.76, 2.03) | 1.01 (0.66, 1.56) | 1.10 (0.81, 1.51) | 1.12 (0.78, 1.61) |
| FEV_1_ / FVC < 0.8 | 1.19 (0.81, 1.74) | 0.70 (0.49, 1.00) | 0.74 (0.50, 1.09) | 0.67 (0.43, 1.03) | 0.91 (0.62, 1.32) | 0.86 (0.66, 1.11) | 0.85 (0.63, 1.15) |
| FEF _25-75_ < LLN* | 1.01 (0.68, 1.45) | **0.65 (0.44, 0.95)** | 0.90 (0.60, 1.35) | 0.71 (0.45, 1.12) | 1.04 (0.71, 1.51) | 0.88 (0.67, 1.14) | 0.88 (0.65, 1.20) |
| FENO > 15 ppb | 1.05 (0.80, 1.39) | 1.15 (0.88, 1.49) | **1.36 (1.02, 1.81)** | **1.42 (1.01, 1.92)** | 1.10 (0.79, 1.54) | 0.99 (0.79, 1.26) | 0.98 (0.74, 1.29) |
| FENO > 35 ppb | 1.20 (0.75, 1.92) | 1.02 (0.67, 1.55) | 1.45 (0.90, 2.32) | 1.23 (0.75, 2.17) | 1.24 (0.75, 2.05) | 0.93 (0.67, 1.30) | 0.93 (0.62, 1.38) |

**Table S7: Logistic regression models showing adjusted associations between pesticide metabolites, lung function indices, and airway inflammation in atopic children at baseline and follow-up.**

|  | **Baseline** | | | | **Follow-up** | | |
| --- | --- | --- | --- | --- | --- | --- | --- |
|  | DEP | DMP | DMPT | ∑DAP | DEP | DMP | ∑DAP |
| FEV_1_ < LLN* | 1.35 (0.88, 2.11) | 0.94 (0.64, 1.38) | 1.03 (0.66, 1.62) | 1.09 (0.66, 1.81) | 0.74 (0.45, 1.22) | 0.91 (0.63, 1.03) | 0.85 (0.56, 1.29) |
| FVC < LLN* | 1.27 (0.73, 2.19) | 1.76 (0.98, 3.17) | 1.61 (0.86, 3.02) | **2.15 (1.04, 4.45)** | 0.81 (0.44, 1.50) | 0.98 (0.64, 1.51) | 0.95 (0.57, 1.57) |
| FEV_1_ / FVC < 0.8 | 0.99 (0.65, 1.52) | 0.72 (0.49, 1.06) | **0.63 (0.39, 0.99)** | 0.63 (0.38, 1.04) | 0.70 (0.43, 1.14) | 0.78 (0.55, 1.10) | 0.71 (0.47, 1.07) |
| FEF _25-75_ < LLN* | 1.12 (0.76, 1.64) | 0.94 (0.64, 1.35) | 0.96 (0.63, 1.48) | 0.97 (0.60, 1.56) | 0.69 (0.42, 1.14) | 0.85 (0.59, 1.21) | 0.76 (0.51, 1.17) |
| FENO > 15 ppb | 1.07 (0.78, 1.47) | 1.09 (0.81, 1.47) | 0.98 (0.71, 1.37) | 1.10 (0.76, 1.61) | 1.00 (0.64, 1.58) | 0.99 (0.69, 1.42) | 0.96 (0.63, 1.45) |
| FENO > 35 ppb | 1.15 (0.78, 1.71) | 0.85 (0.59, 1.22) | 0.99 (0.67, 1.49) | 0.91 (0.58, 1.43) | 1.29 (0.79, 2.12) | 1.05 (0.73, 1.53) | 1.06 (0.69, 1.64) |

**Table S8: Logistic regression models showing adjusted associations between pesticide metabolites, lung function indices, and airway inflammation in non-atopic at baseline and follow-up.**

|  | **Baseline** | | | | **Follow-up** | | |
| --- | --- | --- | --- | --- | --- | --- | --- |
|  | DEP | DMP | DMPT | ∑DAP | DEP | DMP | ∑DAP |
| FEV_1_ < LLN* | 1.14 (0.79, 1.62) | 1.19 (0.85, 1.65) | 1.26 (0.88, 1.79) | 1.24 (0.83, 1.87) | 0.98 (0.68, 1.41) | 0.85 (0.66, 1.11) | 0.86 (0.64, 1.16) |
| FVC < LLN* | 1.12 (0.74, 1.69) | 1.27 (0.86, 1.87) | **1.89 (1.23, 2.91)** | **1.68 (1.03, 2.75)** | 1.14 (0.76, 1.73) | 0.85 (0.62, 1.16) | 0.86 (0.59, 1.22) |
| FEV_1_ / FVC < 0.8 | 1.23 (0.82, 1.82 | 1.18 (0.80, 1.72) | 1.18 (0.79, 1.77) | 1.18 (0.74, 1.89) | 1.03 (0.71, 1.49) | 1.11 (0.84, 1.45) | 1.12 (0.82, 1.54) |
| FEF _25-75_ < LLN* | 1.40 (0.96, 2.03) | 1.08 (0.78, 1.48) | 1.16 (0.82, 1.63) | 1.18 (0.79, 1.78) | 0.99 (0.69, 1.41) | 0.94 (0.73, 1.22) | 0.95 (0.70, 1.27) |
| FENO > 15 ppb | **1.44 (1.05, 1.97)** | 1.02 (0.78, 1.34) | 1.11 (0.83, 1.49) | 1.23 (0.87, 1.75) | 0.78 (0.55, 1.11) | 0.98 (0.77, 1.26) | 0.92 (0.69, 1.22) |
| FENO > 35 ppb | 1.41 (0.32, 6.19) | 2.01 (0.44, 9.17) | 0.65 (0.13, 2.18) | 1.34 (0.22, 7.97) | 0.86 (0.29, 2.49) | 0.69 (0.31, 1.56) | 0.63 (0.25, 1.62) |

**
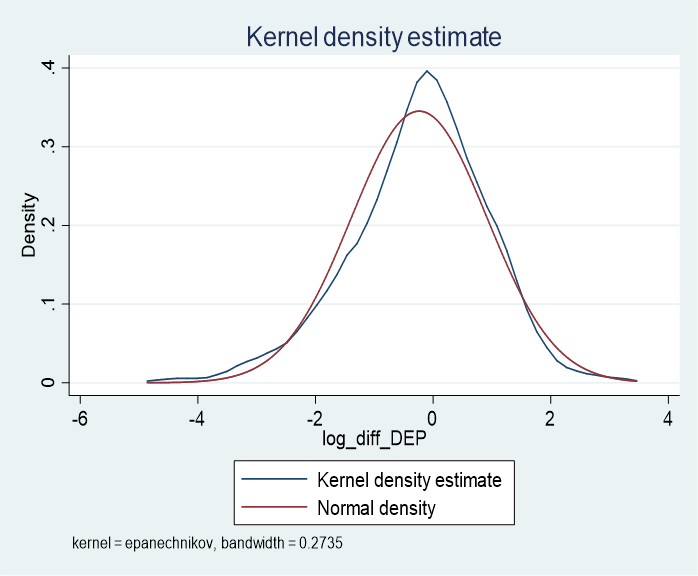

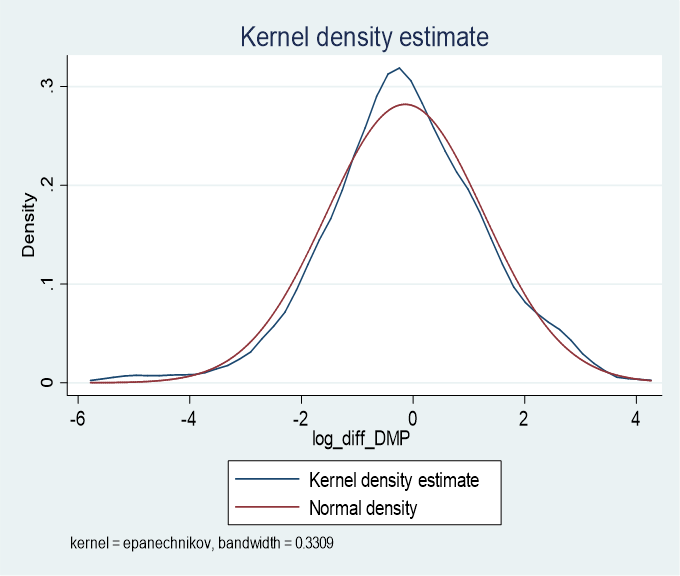
**

**Figure S1: Difference distribution plot of DMP (left) and DEP (right) metabolites, computed from the difference at baseline and follow-up, showing relatively similar concentrations at both study phase.**
